# An enhanced photosynthesis and carbohydrate metabolic capability contributes to heterosis of the cotton (*Gossypium hirsutum*) hybrid ‘Huaza Mian H318’, as revealed by genome-wide gene expression analysis

**DOI:** 10.1186/s12864-021-07580-8

**Published:** 2021-04-17

**Authors:** Yuanhao Ding, Rui Zhang, Longfu Zhu, Maojun Wang, Yizan Ma, Daojun Yuan, Nian Liu, Haiyan Hu, Ling Min, Xianlong Zhang

**Affiliations:** 1 National Key Laboratory of Crop Genetic Improvement, Huazhong Agricultural University, Wuhan, 430070 China; 2 Hainan Key Laboratory for Sustainable Utilization of Tropical Bioresource, College of Tropical Crops, Hainan University, Haikou, 572208 China

**Keywords:** Cotton, Heterosis, RNA-Seq, Photosynthesis, Carbohydrate

## Abstract

**Background:**

Heterosis has been exploited for decades in different crops due to resulting in dramatic increases in yield, but relatively little molecular evidence on this topic was reported in cotton.

**Results:**

The elite cotton hybrid variety ‘Huaza Mian H318’ (H318) and its parental lines were used to explore the source of its yield heterosis. A four-year investigation of yield-related traits showed that the boll number of H318 showed higher stability than that of its two parents, both in suitable and unsuitable climate years. In addition, the hybrid H318 grew faster and showed higher fresh and dry weights than its parental lines at the seedling stage. Transcriptome analysis of seedlings identified 17,308 differentially expressed genes (DEGs) between H318 and its parental lines, and 3490 extremely changed DEGs were screened out for later analysis. Most DEGs (3472/3490) were gathered between H318 and its paternal line (4–5), and only 64 DEGs were found between H318 and its maternal line (B0011), which implied that H318 displays more similar transcriptional patterns to its maternal parent at the seedling stage. GO and KEGG analyses showed that these DEGs were highly enriched in photosynthesis, lipid metabolic, carbohydrate metabolic and oxidation-reduction processes, and the expression level of these DEGs was significantly higher in H318 relative to its parental lines, which implied that photosynthesis, metabolism and stress resistances were enhanced in H318.

**Conclusion:**

The enhanced photosynthesis, lipid and carbohydrate metabolic capabilities contribute to the heterosis of H318 at the seedling stage, and establishes a material foundation for subsequent higher boll-setting rates in complex field environments.

**Supplementary Information:**

The online version contains supplementary material available at 10.1186/s12864-021-07580-8.

## Background

Heterosis is a mysterious and widespread biological phenomenon that has been scientifically investigated but few explicit descriptions of its molecular basis have been documented [1]. Heterosis is extremely prevalent in plants and animals but exhibits differ among developmental stages in different species and across different traits [2]. Moreover, heterosis has benefited production for over a century, causing dramatic improvements in crop yield; some major crops exhibit heterosis in production, such as rice, maize and rapeseeds [3].

Despite the lack of a unified theory, several valuable hypotheses have been presented and widely accepted by biologists [4]. Dominance and overdominance are two classical genetic explanations, especially for single-gene or single-trait heterosis [5, 6]. Additionally, studies in rice over the last 20 years have developed two different genetic models for heterosis: epistasis and pseudodominance [7, 8]. A common opinion about heterosis is that the genetic diversity of the inbred lines from F1 hybrids is a key factor in heterosis performance. In Arabidopsis, the genetic distance of the inbred parents is proportional to the hybrid vigor [9]. However, polyploid hybrids always exhibit higher heterosis than diploid hybrids, although crosses between genetically similar rice lines can also produce hybrids with significant heterosis [10, 11]. Thus, the hypotheses are insufficient in explaining all of the evidence regarding heterosis [2]. The development of a perfect unified theory to explain heterosis is difficult because of the genetic complexity and genomic diversity of different species.

In past years, many transcriptome studies have provided insights into the molecular basis of heterosis in different species, such as rice [8, 12], Arabidopsis [13, 14] and maize [15, 16]. In these studies, an extremely high number of differentially expressed genes (DEGs) associated with different biochemical pathways have been found to be related to heterosis. However, few specific biological pathways have been demonstrated to play key roles in heterosis, and DEGs show a random distribution among biochemical pathways. In addition, genome-wide changes in gene expression have displayed additive or nonadditive effects in different studies and crops [9], which mean that the differing gene actions in hybrids are related to the genetic distance of the parents [17]. Moreover, genome-wide studies of transcriptomes identify which biological pathways are changed in hybrids, including energy, metabolism and biomass, phytohormone signaling and stress responses pathways [18], which contribute to our understanding of heterosis.

Cotton (*Gossypium spp.*) is an important cultivated crop due to the economic value of its fiber. The acreage of cotton cultivation in China has continued to decrease in recent years. The challenge in China is to use less land to produce more cotton to preserve acreage for producing urgently demanded grains. Hybrid cotton is chosen to maintain total cotton production capability with less land. As early as 1894, it was reported that hybrids between *Gossypium hirsutum* L. and *Gossypium barbadense* L. exhibited great heterosis in vegetative growth [19]. Subsequently, several attempts were made to apply similar principles into intraspecific cottons [20, 21]. Previous studies have shown that heterosis in cotton may be correlated with vegetative heterosis at the seedling and squaring stages [22]. During flowering, environmental influences were shown to be correlated with the final yield of a cotton hybrid [23]. Although increasing cotton heterosis research data have accumulated, we are still far from a systematic understanding of heterosis.

H318, a released intraspecific hybrid cotton variety (*Gossypium hirsutum*) derived from a cross between B0011 and 4–5, has been widely adopted for cotton production in southern China due to its high yield and wide range of adaptations. The hybrid and its parents were employed to investigate heterosis performance by comparing genome-wide gene expression profiles by RNA-Seq technology and physiological analysis. A large number of DEGs were found to be involved in photosynthesis, lipid metabolic, carbohydrate metabolic and oxidation-reduction processes. Moreover, the assessment of physiological rate, sugar content and gene expression related to photosynthesis and carbohydrates supported the findings from RNA-Seq. Thus, the enhanced photosynthesis and carbohydrate metabolic capability were considered to contribute to the heterosis performance of H318 at the seedling stage, which might help to elucidate the material foundation of the hybrid’s high adaption to complex field environments and ultimately benefit its higher boll setting rates in different years.

## Results

### Increased boll number contributes to the yield heterosis of H318 in different years

H318, as an elite cotton cultivar, showed an obvious yield increase over the control variety, with an average 3664.5 kg/ha seed cotton and 1521 kg/ha lint yield production (9.2% higher than control) in 2 years (2007–2008) of regional testing.

**Fig. 1:**
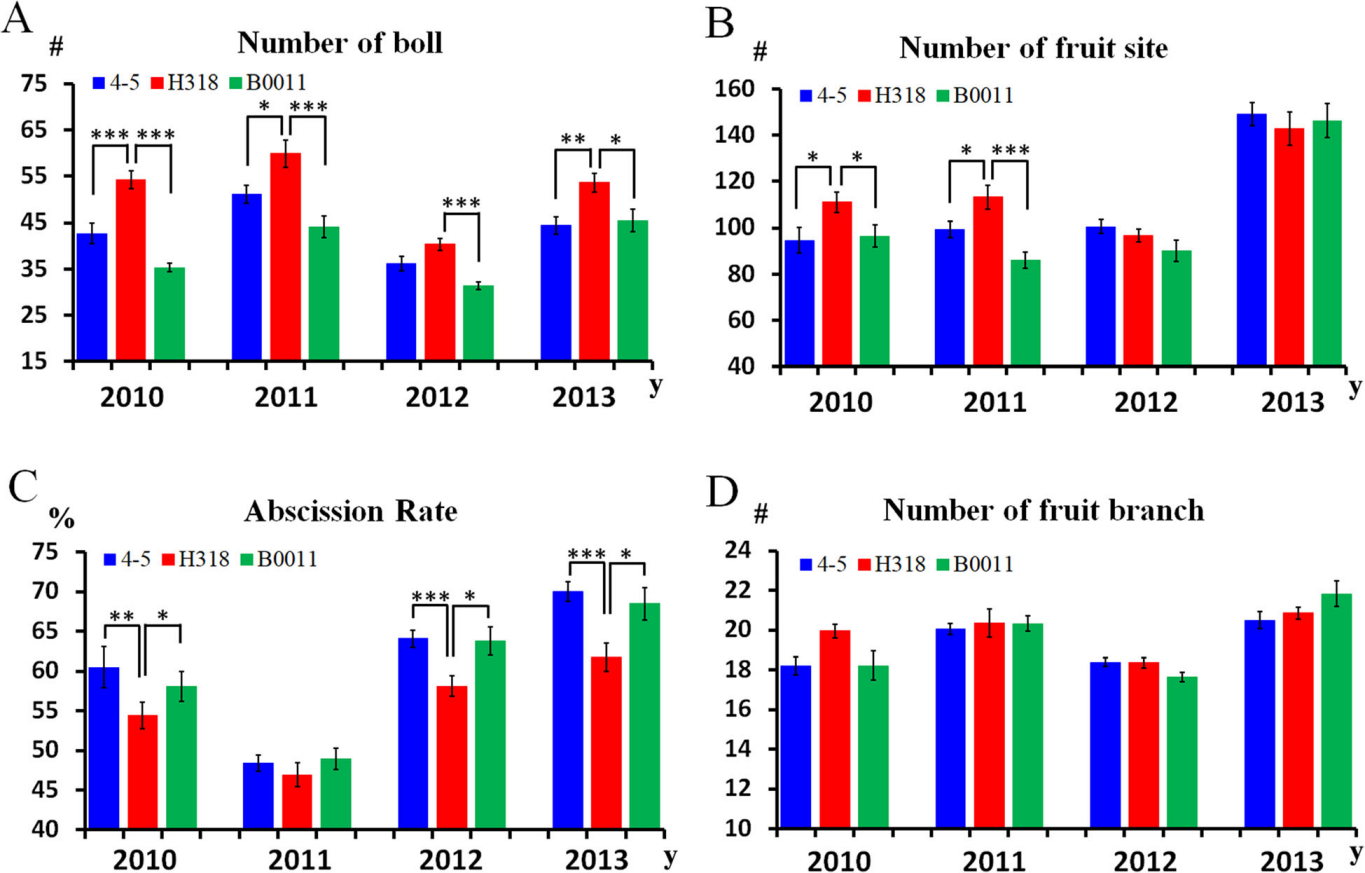
Four years of investigation of yield-related traits in H318 and its parental lines. **a** The number of bolls was stably higher in the hybrid over 4 years. **b** Boll-node number increased in the first 2 years. **c** The abscission rate decreased in 3 out of the 4 years, corresponding to the number of bolls. **d** The average number of fruit branches showed no difference between hybrid and its parents. Asterisks indicate statistically significant differences (* *P* < 0.05, ** *P* < 0.01, *** *P* < 0.001) by Student’s *t* test. The x axis represents year, and the y axis represents numbers or ratios

To explore the effects of heterosis on yield, we conducted a four-year field investigation of traits such as the number of bolls, the number of fruit sites, the abscission rate and the number of fruit branches (Fig. 1a-d). At the same time, considering the climate influence on plant growth and production, the climatic changes from May to October (the whole growth period of cotton) in Wuhan from 2010 to 2013 were also obtained from the Statistics Bureau of Hubei Province (Figure S1 A-D).

In 2010, a long rainfall period and a large volume of precipitation in July (Figure S1 C, D) strongly affected the fruit setting efficiency (July and August are the flourishing flowering stage), which caused high abscission rates in 4–5 (paternal line) and B0011 (maternal line) (Fig. 1c). However, a relatively lower abscission rate and more fruit sites were found in H318 (Fig. 1b-c), which might be the reason for its final higher boll numbers (Fig. 1a).

In later relatively ideal climate conditions in 2011, there was more water availability in the growing stage (June in Figure S1 C, D); suitable sunshine hours, rain and warming temperatures during the flourishing flowering stage (July and August in Figure S1 A-D); and little difference could be found in the abscission rates between the hybrid and the two parents (Fig. 1c). However, many more fruit sites were produced in hybrid H318 (Fig. 1b), which directly caused the increased boll number of H318 (Fig. 1a).

In 2012 and 2013, long-term high temperature stress that occurred in July and August (Figure S1 A) caused a higher abscission rate than the other years (Fig. 1c), but H318 still showed a much lower abscission rate than its parents, and more bolls were produced (Fig. 1a, c). No obvious differences were found in the fruit site in 2012 and 2013, and fruit branches showed no difference in any year (Fig. 1b, d). Thus, we speculated that the higher yield production of H318 directly derived from the stable higher number of bolls, which is the result of increased fruit sites or decreased abscission rate in different years.

### Hybrid H318 shows heterosis of biomass and the growth rate at the seedling stage

**Fig. 2:**
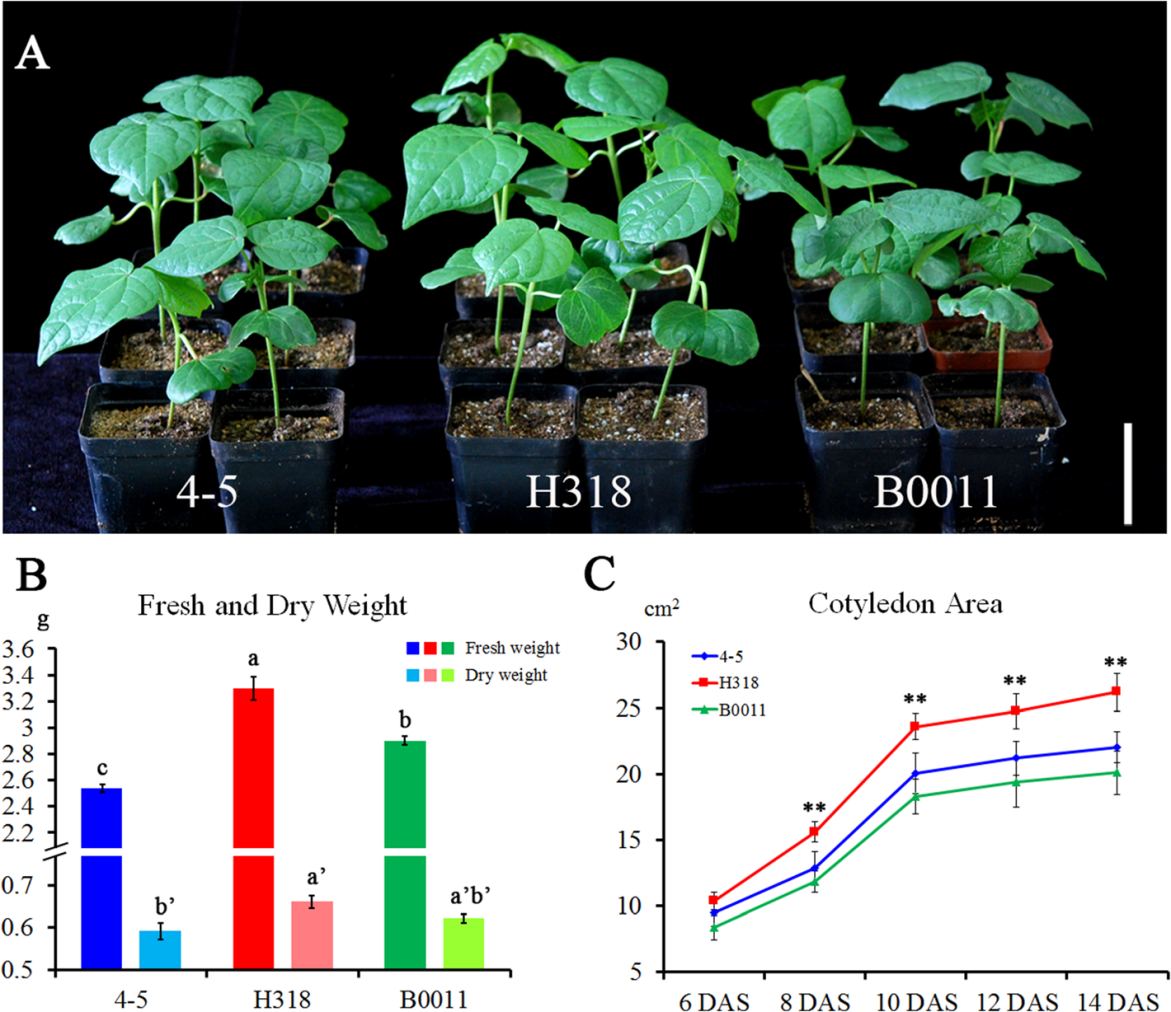
Heterosis performance of H318 at the seedling stage. **A** Phenotype of H318 and its parental lines at the seedling stage. H318 showed obvious growth heterosis at the seedling stage relative to its parental lines. Bar = 5 cm. **B** Fresh and dry weight of H318 and its parental lines. Values with different letters are considered statistically significant (shortest significant range; *P* < 0.05). **C** Cotyledon area of H318 and its parental lines from the 6th to 14th day after sowing (DAS). Asterisks indicate statistically significant differences (* *P* < 0.05, ** *P* < 0.01, *** *P* < 0.001) by Student’s *t* test

In addition to the yield heterosis, H318 also showed growth vigor compared with its parents at the seedling stage (Fig. 2A). The fresh and dry weights (the whole plant) of H318 and its parents were measured at the two-leaf stage, and the results showed that H318 had obviously higher fresh weight than its parents and dry weight than 4–5 (Fig. 2B). Moreover, the fresh weight of B0011 was higher than 4–5, but without obvious changes in dry weight. To evaluate the plant growth status in detail, the cotyledon area was calculated every 2 days after cotyledons spread until 14 days after sowing (DAS) (Fig. 2C). At 6 DAS, hybrid H318 had a slightly larger cotyledon area than two parents. After 8 DAS, the cotyledon area significantly increased and reached its maximum area at 14 DAS. The cotyledon area of H318 remained remarkably larger than its parents after 8 DAS. These results suggested that H318 exhibited obvious heterosis of biomass production and the growth rate at the seedling stage.

### Global analysis of differential gene expression in H318 and its parents

To analyze the global gene expression patterns of these three genotypes, a total of 9 RNA sequencing libraries (containing 3 independent biological repeats for each genotype) were constructed for Illumina sequencing using whole seedlings at 8 DAS. In total, at least 43,000,000 clean reads (accounting for 95% of raw reads) were generated from each sample (Table S1). All clean reads were then mapped to the cotton genome (*G. hirsutum* TM-1 (AD)1). Nearly 89% reads were mapped to the genome, and more than 80% reads were uniquely mapped (Table S2). Most mapped reads (> 85%) were located in exons; reads in intergenic regions occupied 11–12%, and the rest (2.5%) were located in introns (Figure S2).

The reads mapped to the genome were then used for transcript assembly and gene expression level calculation. The expected number of fragments per kilobase of transcript sequence per million base pairs sequenced (FPKM) of all genome annotated genes (70,478) and novel genes (6564) are listed in Table S3. The correlation analysis showed perfect consistency between biological repeats (Figure S3). All 3 possible comparisons were used for differentially expressed gene (DEG) screening with a *P*-value < 0.05. Finally, a total of 17,308 DEGs containing 16,047 annotated genes and 1261 novel genes were found between three genotypes (Table S4), and many few DEGs (306) between H318 and B0011 were found (Figure S4).

**Fig. 3:**
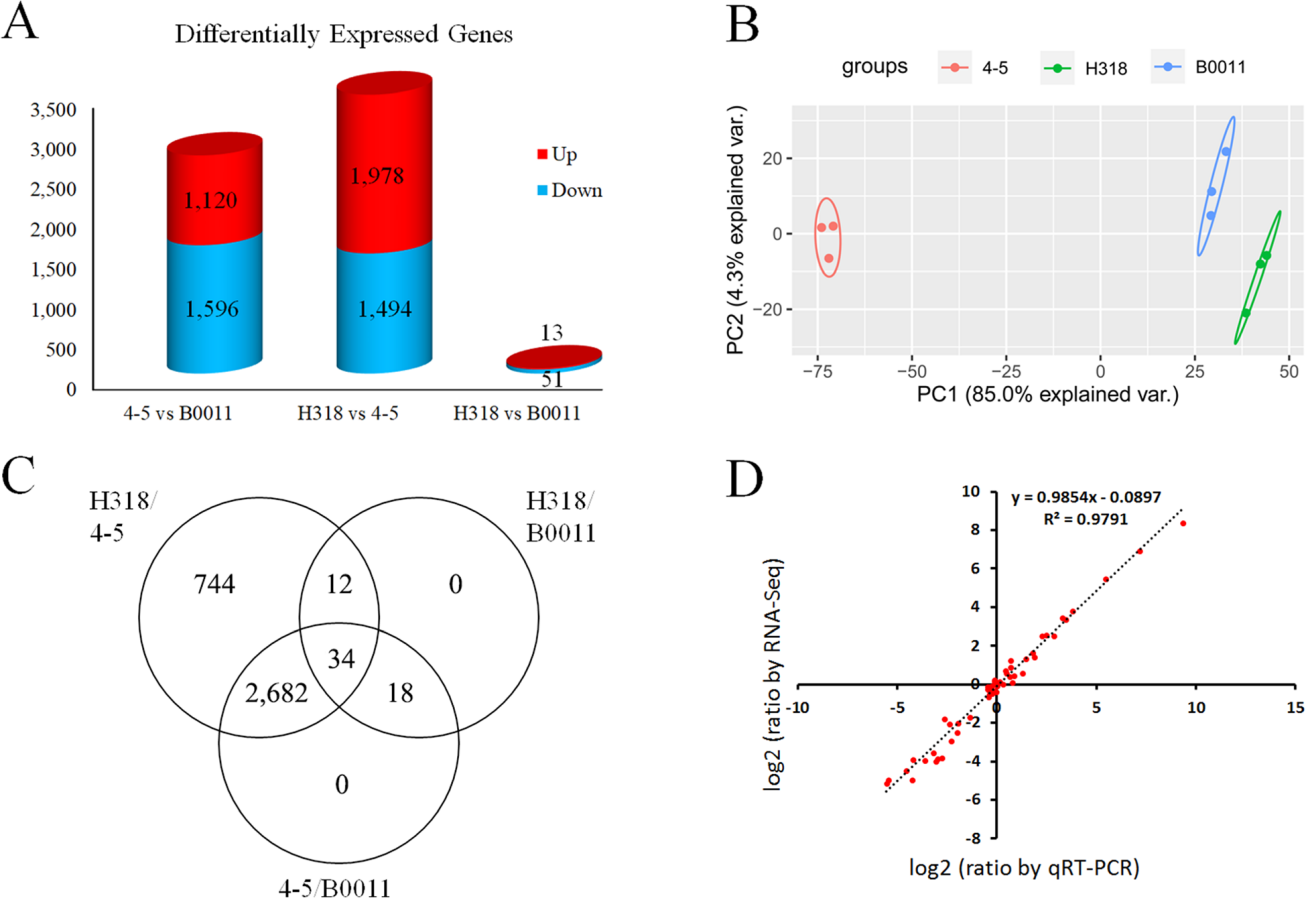
Statistical analysis and qRT-PCR validation of differentially expressed genes (DEGs) from RNA sequencing data. **a** Up- and downregulated DEGs between each sample. **b** PCA analyze with filtered DEGs. **c** The distribution of all DEGs in the three genotypes. **d** Correlation analysis between qRT-PCR results and data from RNA sequencing

To remove the DEGs with low expression or differences, a more stringent criterion (*P*-value < 0.05 and |log_2_Ratio (FPKM+ 1.5)| > 1 in at least one sample) was applied, leaving 3490 DEGs. The FPKM values of the DEGs mostly ranged from 3 to 15 indicating that most DEGs were relatively low expressed (Figure S5), which implied low expressed DEGs were the important part for heterosis behaviour at the seedling stage. In addition, 3472 DEGs were found between H318 and its paternal line (4–5), most DEGs (1978) were upregulated in H318, and only 64 DEGs were found between H318 and its maternal line (B0011, Fig. 3a). Furthermore, principal component analysis (PCA) was applied with 3490 DEGs to show the difference in gene expression between samples (Fig. 3b). The results showed that 3 genotypes were obviously divided from each other, with 4–5 located far from H318 and B0011, indicating that the expression trends of DEGs in 4–5 were much more different from those in H318 and B0011. The DEG distribution in the three genotypes showed that most DEGs (2682/3490) were differentially expressed between H318 and 4–5 or 4–5 and B0011; 34 DEGs were shared in the three genotypes (Fig. 3c). These results indicated that the gene expression pattern of H318 is more similar to that of its maternal line and different from that of its paternal line.

To validate the reliability of RNA-Seq, 26 DEGs were selected randomly for qRT-PCR confirmation. A correlation analysis was performed using data from RNA-Seq and qRT-PCR (Fig. 3d). The results showed that the *R*^2^ value reaches 0.9791, indicating high quality for RNA-Seq.

### GO and KEGG analysis

**Fig. 4:**
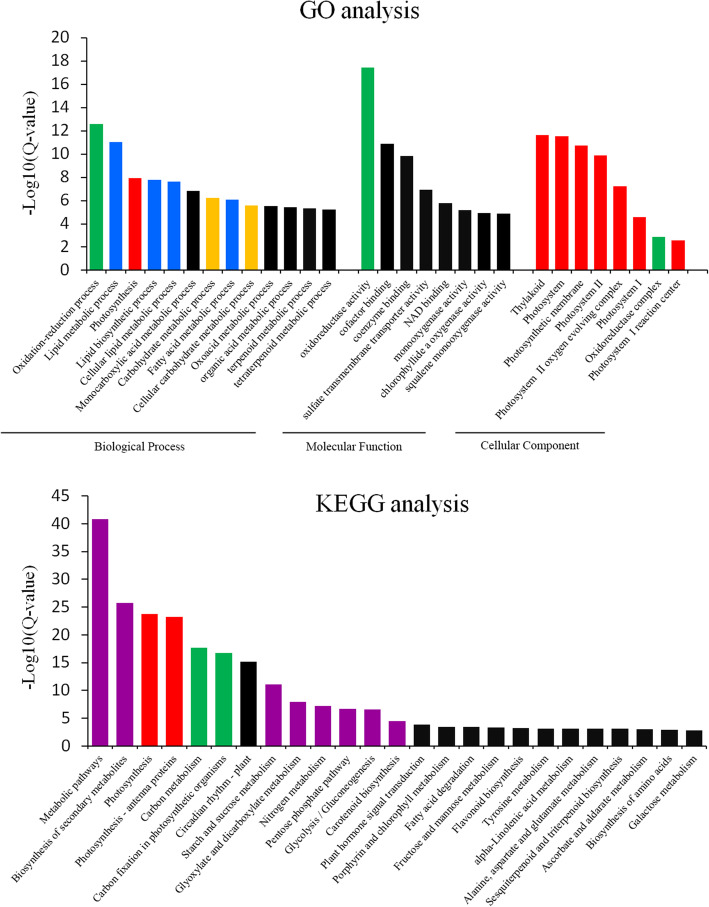
GO and KEGG analysis of DEGs. All processes and pathways were ranged according to the value of –log_10_(q-value)

To gain a deeper understanding of the potential functions of these DEGs (3490) in the three genotypes, GO and KEGG analyses were carried out for gene functional classification (Fig. 4, Tables S5 and S6). For GO analysis, the most significantly enriched biological processes were oxidation-reduction (green), lipid metabolic (blue), photosynthesis (red) and carbohydrate metabolism (yellow)-related processes (Fig. 4, Table S5). At the same time, oxidoreductase activity (green) and photosystem-related components (red) were mostly enriched in molecular function and cellular component, respectively. For KEGG analysis, the significantly enriched pathways were various metabolic-related pathways (purple), photosynthesis (red) and carbon metabolism-related pathways (green) (Fig. 4, Table S6).

### Photosynthesis, lipid metabolic, carbohydrate metabolic and oxidation-reduction pathways are enriched in H318

From the above GO and KEGG analyses, we found that photosynthesis (65 DEGs), lipid metabolism (176 DEGs), carbohydrate metabolism (178 DEGs) and oxidation-reduction (371 DEGs) pathways were significantly enriched in H318 (Table S7). To be more specific, most DEGs associated with photosynthesis had no changes (64/65) between H318 and B0011 (maternal line), but upregulated (55/65) in H318 comparing to the 4–5 (paternal line). However, most of them were downregulated in 4–5 (51/65) comparing to the B0011. For lipid (176 DEGs) and carbohydrate (178 DEGs) metabolic processes, over 100 DEGs from each pathway were upregulated in H318 comparing to 4–5, but quite a few (6/176 DEGs for lipid and 4/178 for carbohydrate metabolic processes) were found differentially expressed between H318 and B0011. In addition, 252 DEGs involved in oxidation-reduction process were found upregulated between H318 and 4–5 while 119 DEGs were downregulated. However, most of them were unchanged between H318 and B0011. The results indicated that the photosynthesis pathway was enhanced in H318 and B0011 relative to 4–5, and the lipid metabolic, carbohydrate metabolic and oxidation-reduction processes were enhanced in H318, which implied that hybrid H318 might exhibit a stronger capability for photosynthesis, lipid, carbohydrate metabolism and oxidation reduction than its parents, especially to the paternal line. We speculated that the enhanced capability of these functions might contribute to the faster growth rate and enhanced biomass of H318 at the seedling stage (Fig. 2).

### Higher photosynthesis rates and sugar accumulation were found in H318 at the seedling stage

**Fig. 5:**
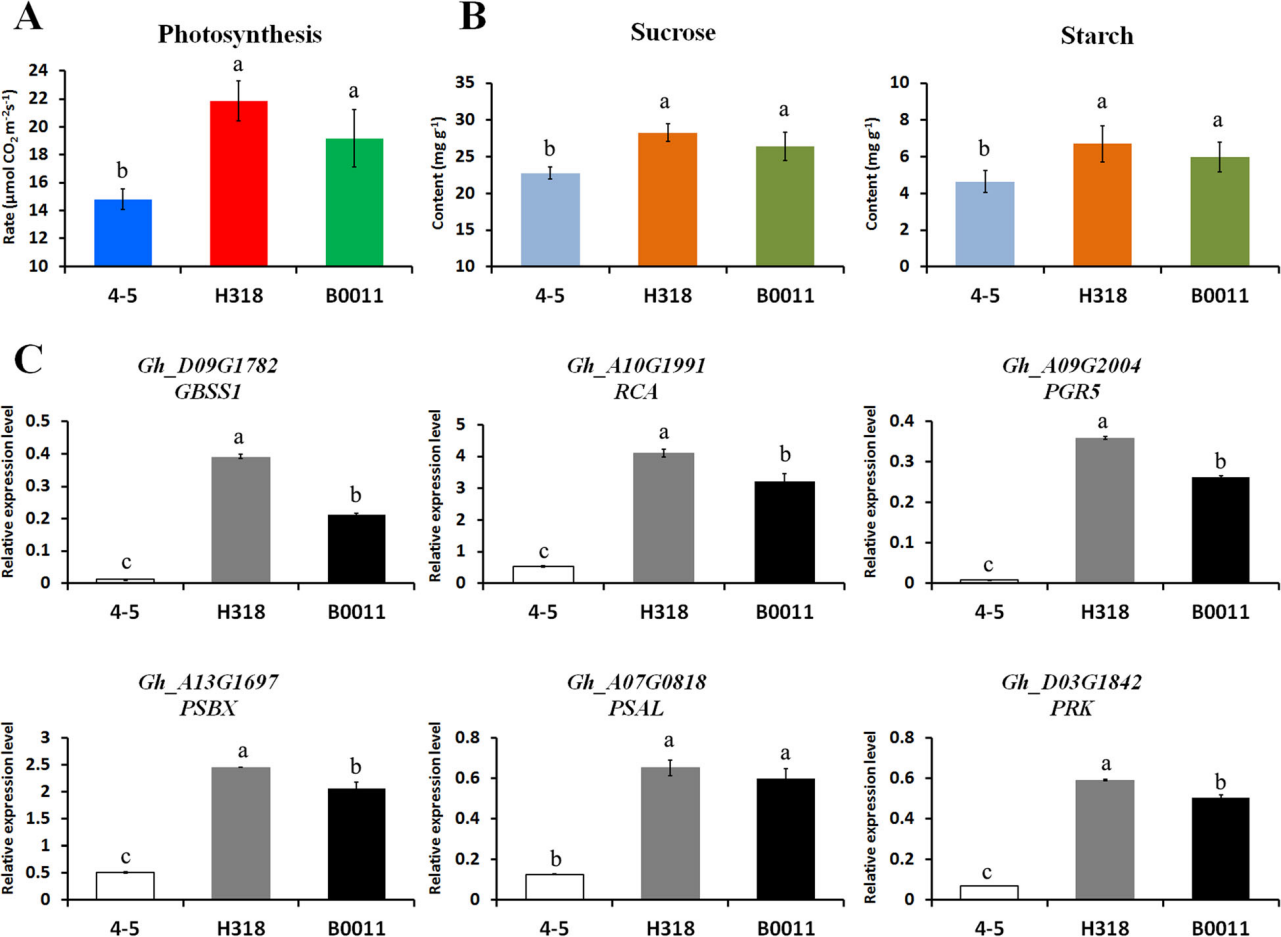
The detection of photosynthesis rates, sucrose, starch and the gene expression changes related to photosynthesis and carbohydrate metabolic processes. **A** Photosynthesis rate of H318 and its parental lines at the seedling stage. **B** The content of sucrose and starch in H318 and its parental lines. **C** Gene expression changes in photosynthesis and carbohydrate metabolic processes. *GhUB7* was used as control. Values with different letters are considered statistically significant (*P* < 0.05)

To validate the authenticity of this inference, the photosynthesis rate and the content of sucrose and starch at the two-leaf stage were assessed in both H318 and its parental lines (Fig. 5). The results showed that the photosynthesis rate was significantly enhanced in H318 relative to its parental lines, especially the paternal line 4–5 (Fig. 5A). Accordingly, the contents of sucrose and starch were also higher in H318 than these in its parental lines (Fig. 5B). Moreover, the relative expression levels of 6 genes related to starch biosynthesis and photosynthesis processes were detected and shown in Fig. 5C, including *GRANULE BOUND STARCH SYNTHASE 1* (*GBSS1*), *RUBISCO ACTIVASE* (*RCA*), *PROTON GRADIENT REGULATION 5* (*PGR5*), *PHOTOSYSTEM II SUBUNIT X* (*PSBX*), *PHOTOSYSTEM I SUBUNIT L* (*PSAL*) and *PHOSPHORIBULOKINASE* (*PRK*). The expression trends of these genes were highly consistent with the photosynthesis rate and the content of sucrose and starch, which indicated an enhanced photosynthesis rate and sugar biosynthesis in H318 relative to its parental lines. All these results implied that stronger photosynthesis and metabolic processes contribute to heterosis in H318 at the seedling stage.

## Discussion

To produce more cotton using less land, heterosis application seems imperative for improving the yield output per unit in China. Differential gene expression studies between hybrids and their parents have confirmed heterosis-related effects in other crops, but little is known about cotton [11, 15]. In this study, we used RNA-Seq technology to analyze the gene expression differences between the high-yield cotton hybrid variety ‘Huaza Mian H318’ and its parents to illustrate the effects of heterosis in cotton.

### A higher boll-setting rate contributes to the yield heterosis of H318

From our yield investigation results, higher boll numbers were thought to be the source of the yield heterosis performance of the hybrid H318 (Fig. 1). Interestingly, H318 stably produced more bolls than its parental lines in four consecutive years under optimal or unsuitable climate conditions. Therefore, we speculated that the stable higher boll setting rate of H318 might be derived from its potential capability in terms of balancing the tradeoff between stress responses and growth. On the other hand, in the hot summer of 2013, the paternal line (4–5) of H318 was more sensitive to high temperature and showed more serious male sterility than H318 and B0011 (Figure S6), which implied that the stress tolerance of H318 might be inherited from its maternal line (B0011).

In past years, transcriptome studies of heterosis have shown that many stress response biological processes were enriched in F1 hybrids in different species [24–26]. In maize, the drought stress tolerance gene *ZAR1* was found to benefit yield heterosis performance [27]. Some TCA cycle intermediates have been reported to contribute to the heterotic phenotype under freezing stress [28], and soluble sugar and flavonol contents have also been shown to present a strong relationship with heterosis performance [29]. A viewpoint in which balancing the tradeoff between a rapid requirement for stress responses and long-term maintenance of growth vigor is balanced was proposed [30]. Thus, we thought that comprehensive stress tolerance and growth vigor both benefit yield heterosis performance and change with circumstances.

### Enhanced photosynthesis, lipid and carbohydrate metabolism resulted in the heterosis of H318 at the seedling stage

In Arabidopsis, various processes or pathways have been reported to be related to the biomass heterosis performance of F1 hybrids, such as photosynthesis, the PIF4-controlled auxin pathway, and stimulus-responsive pathways [31–33]. GA metabolism was found to be related to biomass heterosis in rice seedlings [34]. Our results showed that photosynthesis, lipid and carbohydrate metabolic processes contributed to the hybrid vigor of H318 (Figs. 4 and 5, Table S7). Samples for sequencing were obtained under relatively ideal growth conditions, and no stress response-related pathways exhibited accumulation that seemed natural, although H318 showed high temperature tolerance at the flowering stage. We speculated that increased photosynthesis and energy production was the basis of the biomass heterosis performance of hybrids, and many studies supported this notion [18, 33]. Lipid metabolism has been reported to be enriched in the hybrid rice, oil palm and fish [35–37]. It is widely accepted that gene-gene and gene-environment interactions together determine the final phenotype of a plant. The whole life of a plant can be seen as a process conflicting with various abiotic and biotic stressors from the environment; thus, stress-responsive pathways have been found to be related to heterosis in many studies [31, 38]. However, in a relative suitable growth condition, higher expression of stress-responsive genes will waste large amounts energy; thus, balancing the tradeoff between a rapid requirement for stress responses and long-term maintenance of growth vigor seems like a more economical approach in plants. Therefore, enhanced photosynthesis, lipid and carbohydrate metabolism not only directly result in the biomass heterosis of H318 at the seedling stage but also establish a material foundation for the higher boll-setting rates of H318 at the flowering stage.

In previous years, many important genes or quantitative trait locus (QTLs) associated with heterosis have been identified in many species, such as *WUS* (*WUSCHEL*), *ARGOS* (*AUXIN-REGULATED GENE INVOLVED IN ORGAN SIZE*) in Arabidopsis [31], *SINGLE FLOWER TRUSS* (*SFT*) and *fw2.2* in tomato [39, 40], *GW3p6* (*OsMADS1*), *OsNramp5* (*NATURAL RESISTANCE-ASSOCIATED MACROPHAGE PROTEIN 5*), *Ghd8*/*DTH8*, *Gn1a* (*OsCKX2*), *IPA1* (*OsSPL14*), *Hd3a* (*HEADING DATE3a*) in rice [41–44] and *CNR* in maize [45]. These genes involved in various functions including cell number or cell cycle regulation; environmental adaption; heading date, plant height and grain-yield controlling. To date, few key genes associated heterosis have been found in cotton. In this study, 4 enriched process containing 790 DEGs involved in cotton hybrid vigor at the seedling stage were found (Table S7), which provided the potential important genes resources for heterosis illustration.

## Conclusions

In this study, the elite cotton hybrid variety H318 and its parental lines were used to explore the source of its yield heterosis. Four years of investigation of yield-related traits and transcriptome analysis of seedlings between H318 and its parental lines revealed that photosynthesis and carbohydrate metabolic processes were enhanced in H318, which not only contributed to the heterosis performance of H318 at the seedling stage but also established the material foundation for its higher boll-setting rates in complex field environments over many years.

## Methods

### Plant material, growth conditions and trait investigation

All plant materials used in this study were developed by the Group of Cotton Genetic Improvement (GCGI), Huazhong Agricultural University. Cotton cultivars 4–5 and B0011 (both are *Gossypium hirsutum*) were grown under normal field conditions in Wuhan, and hybrid H318 seeds were developed by hand-pollinating the pollen from 4 to 5 to the emasculated flowers of B0011 before 10 o’clock in the summer. Meanwhile, the two parents were self-pollinated to maintain genetically pure lines.

Seeds of H318 and its parents were sown in mid-April every year to investigate the performance of the traits related to heterosis with 12 plants for each line and 4 lines per plot in a randomized design, repeated in triplicate. Plant spacing was set at 30 cm, and the line spacing was 1 m. Traits such as boll number, plant height, fruit branches and fruit sites were investigated at three time points: July, August and September. In October, 20 bolls from the mid nodes were harvested from the material in each plot to calculate the single boll weight, lint percentage and lint index.

To evaluate heterosis at the seedling stage, cotton seeds were germinated in an incubator at 28 °C for 2 days. They were then transplanted into sand by choosing 30 seedlings with similar radicle lengths to avoid the effects of seed vigor. The plants were watered every 2 days and grown in a controlled growth chamber (16/8 h photoperiod, day and night, at 28 °C, with light intensity of 200 μmol *m*^− 2^ *s*^− 1^) until 14 days after sowing (DAS). Cotyledon areas were measured from 6 DAS to 14 DAS according to previously reported methods [46]. Fresh and dry weights were calculated at 14 DAS; 5 random plants were harvested as one replicate for each type, and 3 replicates were prepared for each material. Another 5 whole plants (aerial and underground parts) for each genotype were harvested and ground in liquid nitrogen immediately for later RNA extraction, and 3 replicates were performed. Photosynthesis was measured with the first expanded true leaf by LI-6400 XT (LI-COR, USA).

### RNA isolation and sequencing library construction

Total RNA was extracted by a modified guanidine thiocyanate method [47] and quantitated by a Nanodrop 2000 instrument (Thermo Scientific, USA). Three total RNA samples per genotype (20 μg for each sample) were sent to Novogene (Beijing, China) for construction of libraries and were then sequenced using the Illumina HiSeq 2500 sequencer.

In brief, the high-quality RNA samples (tested by the Agilent 2100 Bioanalyzer system) were first enriched to obtain mRNA with poly-(A^+^) by using magnetic beads containing OligodT. Then, enriched mRNA was broken into short fragments by fragmentation buffer. The first-strand cDNAs were synthesized using random hexamers and reverse transcriptase. The second-strand cDNAs were generated by *Escherichia coli* polymerase I. The final cDNA libraries were prepared after a round of purification, terminal repair, A-tailing, ligation of sequencing adapters, size selection and PCR enrichment. Completed libraries were then used for sequencing after a series of quality tests.

### Bioinformatics analysis

The original raw data from the Illumina HiSeq2500 sequencer were first filtered to generate clean data by removing reads containing adapters, *N* > 10% or reads with low quality nucleotides > 50%. The clean data were then mapped to the cotton genome (*G. hirsutum* TM-1 (AD)1) [48] to obtain the genome information of each read by TopHat2 [49]. Finally, the reads mapped to genes or exons were used to calculate the gene expression level by HTSeq and shown with FPKM (expected number of fragments per kilobase of transcript sequence per million base pairs sequenced) [50]. Then, the FPKM mean value of 3 biological replications of each variety was calculated representing the gene expression level. DEGs between samples were obtained by screening conditions of P-adj (*P*-value adjusted) < 0.05 and |log_2_Ratio| > 1 (Ratio = FPKM+ 1.5 of sample1/FPKM+ 1.5 of sample 2) for later in-depth analysis.

### GO and KEGG analysis

Gene ontology (GO) term analysis (www.geneontology.org) was applied to predict the functional category distribution frequency of screened DEGs using Blast2GO software. The functional categories with P-adj < 0.05 were kept for later analysis. To understand the differences in pathways, KEGG analysis was also performed with KOBAS 3.0 (http://kobas.cbi.pku.edu.cn/). All pathways were screened according to the criterion of and P-adj < 0.05.

### qRT-PCR analysis

A subset of DEGs (20) was selected to validate the RNA-Seq results through qRT-PCR. Gene-specific primers were designed though Primer Premier 5.0 and synthesized by Genscript Bioscience (Nanjing, China). cDNA was generated from 3 μg RNA samples by Superscript III RT (Invitrogen, Carlsbad, CA) according to the manufacturer’s instructions. qRT-PCR (20 μl) were performed with 9.6 μl of 100× diluted cDNA, 0.2 μl of forward and reverse gene-specific primers, and 10 μl of SYBR Green PCR Master Mix (Applied Biosystems) and then run in four duplicates on an ABI Prism 7500 Sequence Detection System (Applied Biosystems). Thermal cycling conditions were as follows: 30 s at 95 °C, followed by 40 cycles of 5 s at 95 °C and 35 s at 60 °C. Relative quantitation of gene expression was calculated and normalized using GhUBQ7 (GenBank accession number: DQ116441) as an internal standard according to a previous study [51]. Primers used in this study were list in Table S8.

### Sucrose and starch measurement

To validate the changes in photosynthesis and carbohydrate metabolites in the hybrid, approximately 50 mg of plant tissues (the entire plants were fully ground in liquid nitrogen) was obtained from each genotype and incubated in sterile ddH_2_O at room temperature for 24 h for dissolved extraction. The subsequent procedures were performed essentially according to previous reports [52]. The supernatant was used for the measurements of sucrose contents. The remaining pellets were used to assay starch content using an improved colorimetric method [53].

### Statistics

Each graphical plot is generated by the results of multiple independent experiments (*n* ≥ 3), and the values are means ±SE. Statistically significant differences was determined by Student’s *t* tests, and *P* value < 0.05 were considered statistically significant.

## Supplementary Information

**Additional file 1: Figure S1.** The climate changes in summer from 2010 to 2013. Data come from the Statistics Bureau of Hubei Province (http://www.stats-hb.gov.cn/).

**Additional file 2: Figure S2.** Read distribution in the genome.

**Additional file 3: Figure S3.** The correlation analysis between biological repeats.

**Additional file 4: Figure S4.** Volcano plot for differentially expressed genes. The threshold was set as P-adj < 0.05. Red and green plots represent up- and downregulated genes, respectively.

**Additional file 5: Figure S5.** FPKM values distribution of filtered DEGs. Three biological repeats were merged together by the mean values.

**Additional file 6: Figure S6.** The fertility phenotype of H318 and its parental lines under continuous high temperature weather. A: The anthers of 4–5 were indehiscent under high temperature stress, but the anthers of H318 and B0011 dehisced normally. Bar = 1 cm. B: The flowers from H318 and its parental lines were stained by I_2_-KI solution, and the red arrows showed that the anther of 4–5 cannot be stained by I_2_-KI. Bar = 5 mm.

**Additional file 7: Table S1.** Basic quality of sequencing data. **Table S2.** Reads distribution and annotation in genome. **Table S3.** The FPKM value of annotated and novel genes. **Table S4.** All DEGs from RNA sequencing. **Table S5.** Significant GO terms in 945 DEGs screened with condition of *p*-value < 0.05 and |log2(FPKM + 1.5)| > 1. GO terms were listed with gene number ≥ 10, q-value < 0.05. **Table S6.** KEGG pathway analysis of 945 DEGs with q-value < 0.05 using KOBAS3.0 (http://kobas.cbi.pku.edu.cn/). **Table S7.** The expression patterns of DEGs in GO terms ‘oxidation-reduction process’, ‘lipid metabolic process’ , ‘carbohydrate metabolic process’and ‘photosynthesis’. **Table S8.** The primers used in this study.

## Data Availability

The RNA sequencing data used in this study can be found in the National Center for Biotechnology Information (NCBI) SRA database under following accession number: PRJNA393079.
