# Supplementary figures and images for: An enhanced photosynthesis and carbohydrate metabolic capability contributes to heterosis of the cotton (Gossypium hirsutum) hybrid ‘Huaza Mian H318’, as revealed by genome-wide gene expression analysis
Source: BMC Genomics. 2021 Apr 17;22:277. doi: 10.1186/s12864-021-07580-8 (PMC8052695; doi:10.1186/s12864-021-07580-8)

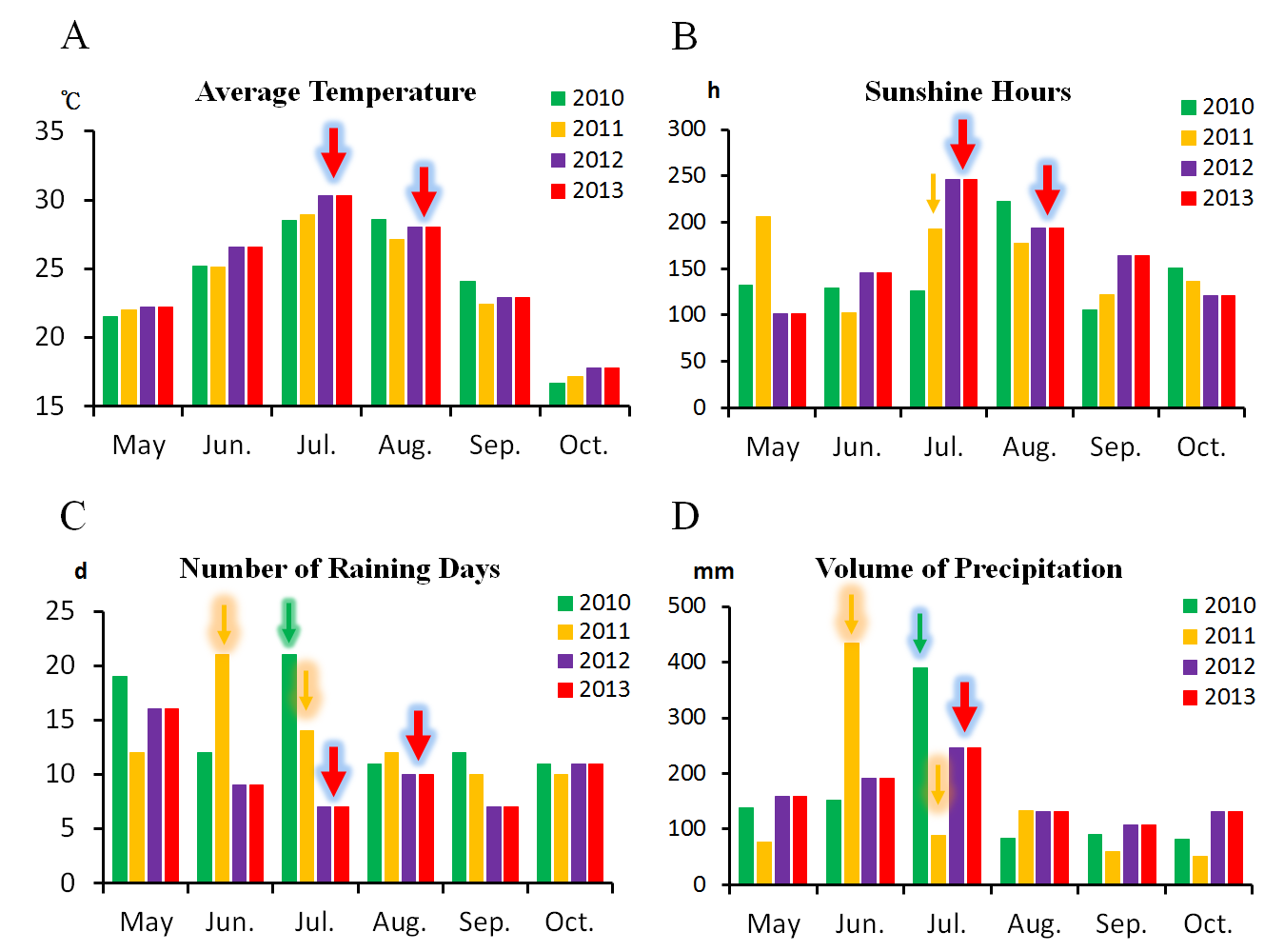

Supplement: Supplementary file 1 — Additional file 1: Figure S1. The climate changes in summer from 2010 to 2013. Data come from the Statistics Bureau of Hubei Province (http://www.stats-hb.gov.cn/). [file 12864_2021_7580_MOESM1_ESM.tif]

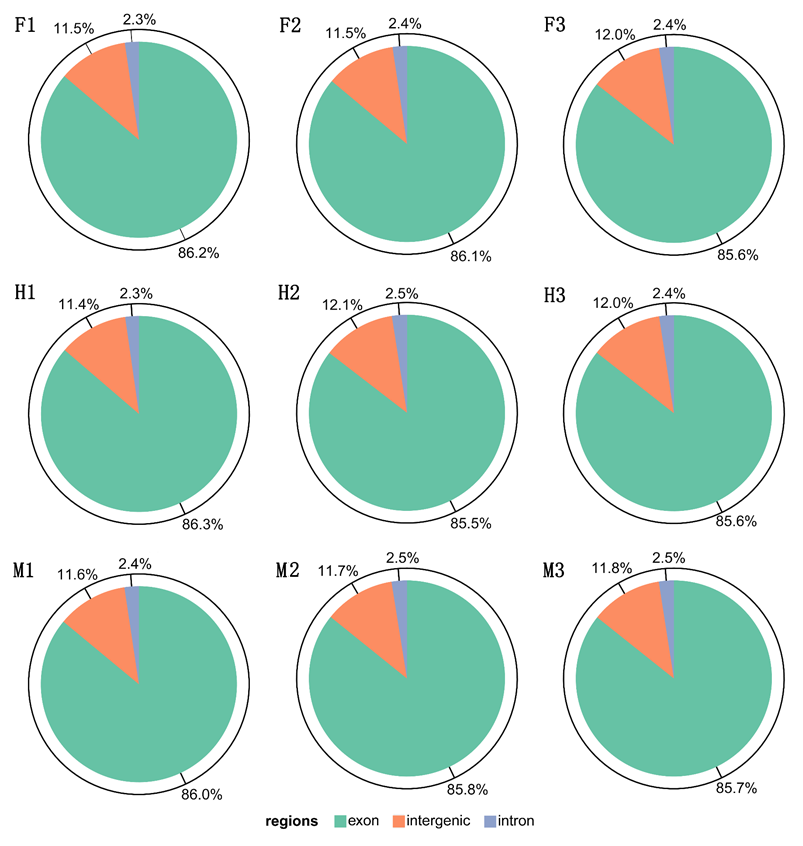

Supplement: Supplementary file 2 — Additional file 2: Figure S2. Read distribution in the genome. [file 12864_2021_7580_MOESM2_ESM.tif]

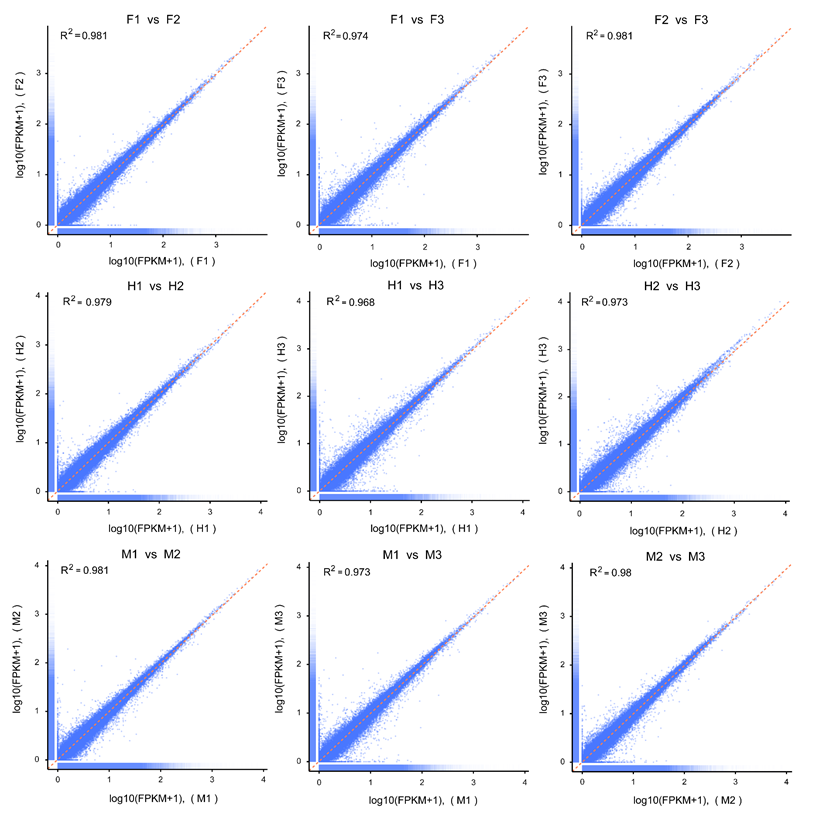

Supplement: Supplementary file 3 — Additional file 3: Figure S3. The correlation analysis between biological repeats. [file 12864_2021_7580_MOESM3_ESM.tif]

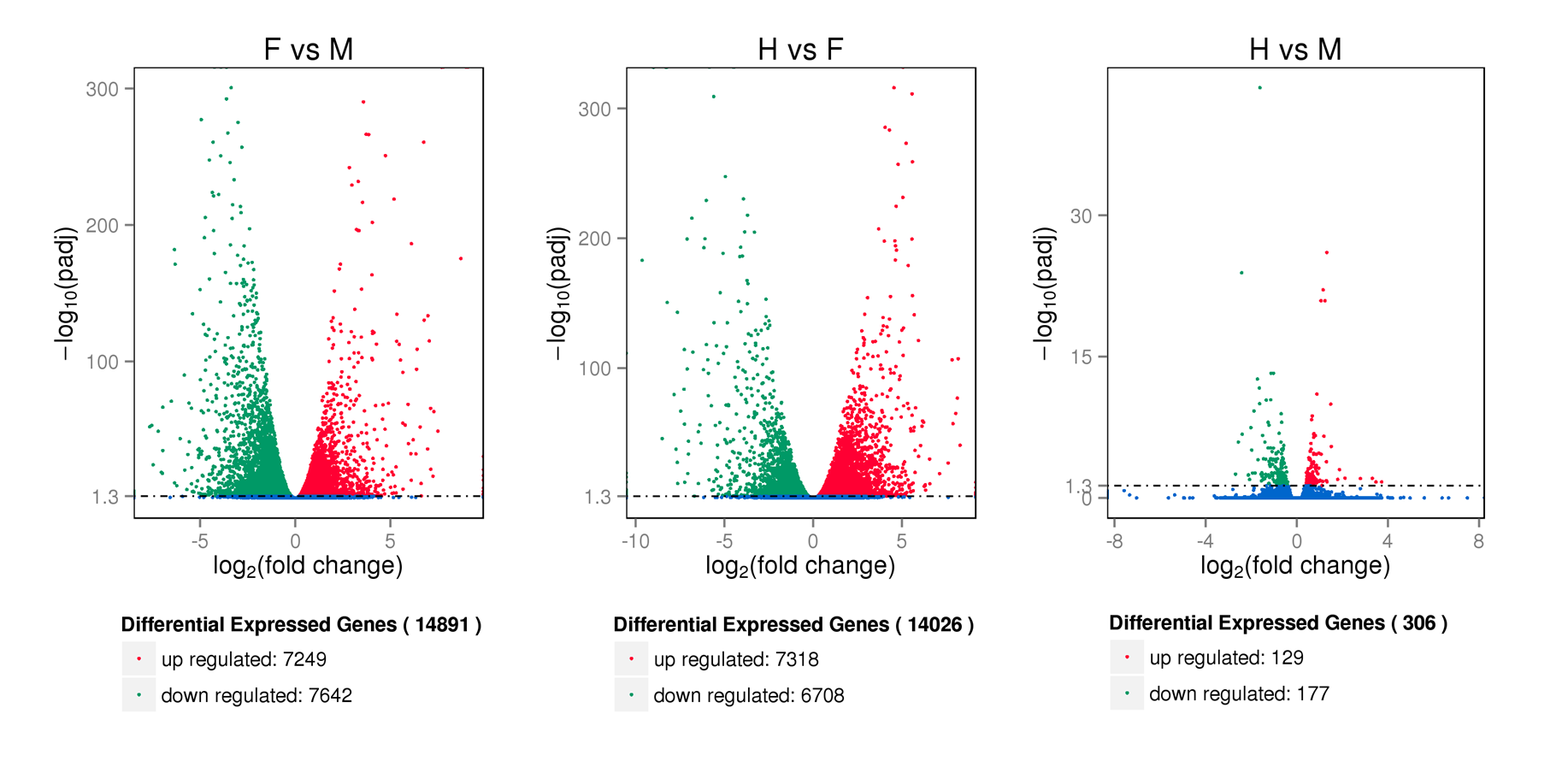

Supplement: Supplementary file 4 — Additional file 4: Figure S4. Volcano plot for differentially expressed genes. The threshold was set as P-adj < 0.05. Red and green plots represent up- and downregulated genes, respectively. [file 12864_2021_7580_MOESM4_ESM.tif]

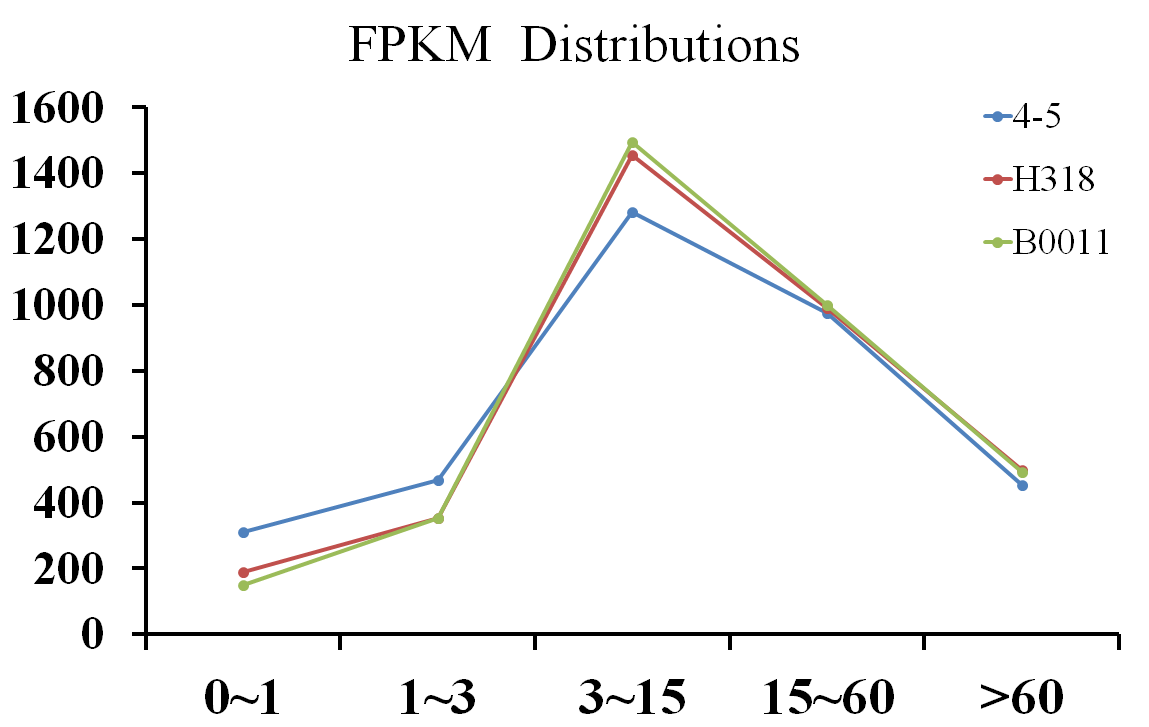

Supplement: Supplementary file 5 — Additional file 5: Figure S5. FPKM values distribution of filtered DEGs. Three biological repeats were merged together by the mean values. [file 12864_2021_7580_MOESM5_ESM.tif]

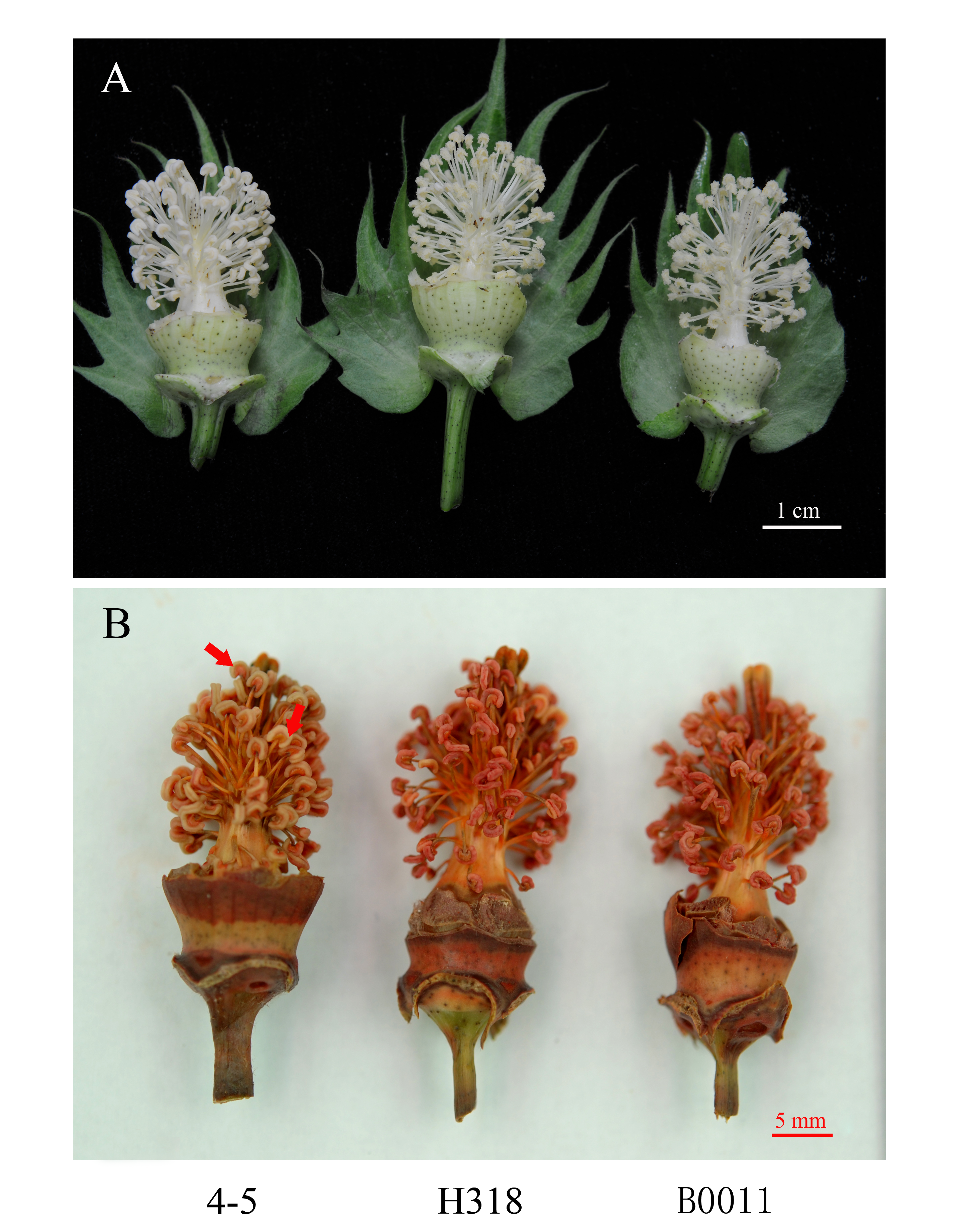

Supplement: Supplementary file 6 — Additional file 6: Figure S6. The fertility phenotype of H318 and its parental lines under continuous high temperature weather. A: The anthers of 4–5 were indehiscent under high temperature stress, but the anthers of H318 and B0011 dehisced normally. Bar = 1 cm. B: The flowers from H318 and its parental lines were stained by I2-KI solution, and the red arrows showed that the anther of 4–5 cannot be stained by I2-KI. Bar = 5 mm. [file 12864_2021_7580_MOESM6_ESM.tif]
